# Insights About Cannabis and Psychosis Using Video Games for Young People With a First Episode of Psychosis, Particularly Those From Black Racialized Communities: Protocol for a Mixed Methods Study

**DOI:** 10.2196/36758

**Published:** 2022-05-20

**Authors:** Suzanne Archie, Lena Palaniyappan, Andrew T Olagunju, Natasha Johnson, Nicole Kozloff, Elham Sadeh, Andrea Bardell, Alexandra Baines, Kelly K Anderson, Oyedeji Ayonrinde, Manuela Ferrari

**Affiliations:** 1 McMaster University Hamilton, ON Canada; 2 Department of Psychiatry Schulich School of Medicine and Dentistry Western University London, ON Canada; 3 Robarts Research Institute Western University London, ON Canada; 4 Lawson Health Research Institute London, ON Canada; 5 Department of Psychiatry and Behavioral Neurosciences McMaster University Hamilton, ON Canada; 6 Discipline of Psychiatry The University of Adelaide Adelaide Australia; 7 Department of Pediatrics McMaster University Hamilton, ON Canada; 8 Slaight Family Centre for Youth in Transition Centre for Addiction and Mental Health Toronto, ON Canada; 9 Department of Psychiatry University of Toronto Toronto, ON Canada; 10 On Track Champlain District Regional First Episode Psychosis Program The Ottawa Hospital Ottawa, ON Canada; 11 Canadian Consortium for Early Psychosis Intervention Ottawa, ON Canada; 12 Department of Psychiatry University of British Columbia Vancouver, BC Canada; 13 Ottawa Hospital Research Institute Ottawa, ON Canada; 14 Department of Psychiatry University of Ottawa Ottawa, ON Canada; 15 Integrated Schizophrenia and Recovery Program The Royal Ottawa Hospital Ottawa, ON Canada; 16 Department of Psychiatry Queen's University Kingston, ON Canada; 17 Department of Psychiatry McGill University Montreal, QC Canada

**Keywords:** first-episode psychosis, cannabis use, knowledge translation, Black youth, video games

## Abstract

**Background:**

Cannabis use disorder among young people with a first episode of psychosis contributes to relapse, hospitalization, and impaired functioning. However, few studies have examined what young people with early phase psychosis, particularly those from Black racialized communities, understand or appreciate about this relationship, even though they may be at risk. There are no formally tested knowledge translation strategies that disseminate these research findings for young people with emerging psychosis from Black racialized communities.

**Objective:**

This study aims to conceptualize what young people with early phase psychosis/cannabis use disorder understand about the relationship between cannabis and psychosis, focusing on people from racialized backgrounds. This study also aims to assess whether the knowledge translation product, the “Back to Reality Series,” increases awareness of the impact of cannabis use on psychosis from the perspectives of young people with emerging psychosis and cannabis use disorder from Black African and Caribbean communities.

**Methods:**

Qualitative analysis will reveal themes from qualitative interviews about cannabis and psychosis from the perspectives of young people with emerging psychosis and cannabis use disorder from Black African and Caribbean communities. Perceptions before and after exposure to the Back to Reality Series will be qualitatively analyzed. A control game will be used for comparison, and scores on a quiz after playing the Back to Reality Series will be quantitatively analyzed to establish whether the Back to Reality Series raises awareness of the effects of cannabis on psychosis. An advisory council involving young people from Black communities, family members, and clinicians will bring community perspectives to this research.

**Results:**

We began recruiting participants for this study in September 2021. We will complete data collection on demographic and clinical factors, qualitative interviews, and quantitative assessments of the Back to Reality Series.

**Conclusions:**

The voices of young people from racialized backgrounds will generate preliminary data to inform early psychosis programs, addressing cannabis use in this population. The findings may advance the use of a new knowledge translation product that deals with gaps in knowledge about cannabis use for people experiencing early phase psychosis, particularly those from racialized communities.

**International Registered Report Identifier (IRRID):**

DERR1-10.2196/36758

## Introduction

### Background

Regular cannabis use (3 times a week or more) is widely recognized as a significant risk factor for relapse of psychosis among young people under 25 years experiencing emerging psychosis [1,2]. Hospitalizations for cannabis-related psychotic disorders in Canada have increased significantly from 2006 to 2015 [3]. Although the association is complex and multi-directional, variables that moderate this relationship include the age of first use [4], the frequency of use [4], the genetic vulnerability for schizophrenia [5-7], the delta-9-tetrahydrocannabinol (THC) content of cannabis [8], and previous psychosis symptoms [4,8]. The higher the THC content, the greater the risk [4,8]. Unfortunately, the people who would benefit the most from this information are often the least likely to know about it [9,10].

Studies suggest that patients experiencing a first episode of psychosis, who used cannabis but stopped, had fewer hospitalizations and had better functioning than those who never used or those who did not stop using [11]. Compared with those who stopped, patients with a first episode of psychosis and ongoing marijuana use were significantly more likely to relapse and experience readmissions [1].

There are no established therapies for cannabis use disorder in patients with a first episode of psychosis [12]. Pharmacotherapies have not yet been shown to be effective in this population [13]. Cognitive behavioral therapy (CBT) and motivational interviewing are not more effective than early psychosis intervention services alone for patients with a first episode of psychosis and cannabis use disorder [13,14]. Early psychosis intervention services provide care to youth and young people experiencing a first episode of psychosis [15], which can significantly reduce substance use [16], but many patients with a first episode of psychosis do not improve their substance use disorders or engage in substance use treatments [16]. Novel strategies are needed to augment the therapy offered by early psychosis intervention programs.

### Insight About Cannabis and Psychosis

To gain insight, young people must first appreciate what psychosis is and the impact cannabis could have on their recovery. Increasing insight about the impact of cannabis use upon relapse might improve outcomes among young people with a first episode of psychosis and cannabis use disorder, primarily if this insight has occurred early in treatment. However, few studies have examined what young people with early phase psychosis understand about this relationship, even though they may be the ones most at risk. Few studies explore the viewpoints of people experiencing a first episode of psychosis on the link between substances of abuse and psychosis, let alone cannabis use. Most qualitative studies focus on people experiencing chronic illness [17]. However, there may be differences in how people with chronic schizophrenia conceptualize the impact of cannabis use on their health compared to those with emerging psychosis [18].

One of the few qualitative studies capturing participants’ perspectives with a first episode of psychosis found a wide variety of beliefs [18]. Some patients drew connections between substances of abuse and the onset of psychosis. In contrast, others were unaware or did not believe there was an association [18]. Young people experiencing a first episode of psychosis reported several benefits of cannabis or other substances. Cannabis and other substances were viewed as social lubricants, enhancing the social milieu they experienced with their friends [19]. They felt it was a pleasurable activity that reduced anxiety, increased creativity, and increased enjoyment of music [19]. However, these young people also expressed concerns about their substance use [19]. This Canadian qualitative study suggested that some participants thought that cannabis use was more likely to trigger psychosis experiences compared with other substances [19]. Moreover, this study indicated that participants believed substances of abuse helped normalize their altered perceptions because their peer group shared time-limited yet similar psychosis-like experiences [19]. However, none of these studies focused specifically on cannabis.

Researchers and service providers need to understand what young people with emerging psychosis believe about cannabis. Understanding their perspectives may support the creation of targeted and effective strategies with the potential to improve their insight and ultimately engage them in cannabis reduction strategies. This need may be particularly poignant for young people with cannabis use disorder and a first episode of psychosis from racialized backgrounds.

### Racialized Groups, Psychosis, and Cannabis

Compared with the native-born population, higher rates of psychosis incidence in Ontario have been reported among Black racialized immigrants to Canada [20]. For Black communities, cannabis use is built on a history of stigma, negative stereotypes, and criminalization [21]. Compared with young people from other ethnic groups, Black youth may be more susceptible to the adverse mental effects of cannabis use because of health and legal inequities. Significantly more patients with a first episode of psychosis and a substance use disorder, including cannabis, were arrested compared with their nonsubstance abusing counterparts, regardless of ethnicity [16]. However, an arrest may have more lasting consequences for young people from Black communities. More specifically, the self-reported rates of cannabis use in Toronto (2018) were higher for nonracialized youth under 19 years (39% nonracialized versus 14% Black). However, about 15% of underage Black youth were detained for bail hearings for cannabis possession. In comparison, only 3% from the comparable nonracialized group [22] experienced this consequence, suggesting underage Black youth experience more punitive legal ramifications when arrested for cannabis. A Canadian study involving adult respondents revealed a significantly higher odds ratio of problematic cannabis use among respondents who self-identified as Black Caribbean compared with the general Canadian population and people of Black African ethnicity [23]. The researchers defined “problematic use” as a pattern of use with a high probability of harm based on the World Health Organization Alcohol, Smoking, and Substance Involvement Screening Tool [23]. Heavy cannabis use appears to be more correlated with youth experiencing psychosis compared with other conditions. Black youth with a first episode of psychosis had a significantly higher frequency of cannabis use and smoked significantly more “joints” than Black youth with other mental health conditions [24]. In summary, these studies highlight the need for early intervention to support Black youth with emerging psychosis. Culturally grounded substance abuse programs exist for Black racialized communities, but these programs do not address psychosis [25]. A “one-size-fits-all” approach may not work for racialized groups who may need more targeted initiatives [26].

### Video Games

Serious game design principles incorporate the following conceptual framework: learning by players, storytelling within the game, interactive gameplay, and user experiences to promote emotional engagement and prosocial attitudes [27]. At present, video games are an underused medium in health care, despite growing evidence supporting their educational effectiveness. A meta-analysis of randomized controlled trials on the effectiveness of serious video games revealed a significant increase in knowledge transfer among youth with chronic conditions compared with control games [28]. Serious video games can be a form of simulation-based training, testing new ideas and skills through interactive play [27,29,30]. Playing video games was ranked as one of the top 10 favorite activities among youth attending an early psychosis intervention program. Over 80% had access to smartphones and computers [31]. Video game technology helps depict psychosis-like experiences. A commercial video game, *Debris*, which was created with input from an industry-academia partnership, weaved psychosis simulations into the gameplay experience [32] as a means to mobilize concepts about psychosis to a general audience. Moonray Studios of Hamilton sold it to PlayStation 4 in 2019. *On Track<The Game* was designed to promote engagement in care among young people who have experienced a first episode of psychosis [33]. Qualitative and quantitative analyses revealed a significant increase in hope, engagement, empowerment, and recovery attitudes at baseline and 2 weeks after playing the game. Participants had an opportunity to use the interactivity to learn about psychosis and practice coping skills [33]. Virtual reality technology significantly reduced auditory hallucinations and paranoia among participants with a first episode of psychosis, when combined with CBT [34]. *Sparx* is a video game that uses avatars to train players to use CBT skills and has been shown to treat depression among adolescents [35]. Finally, successful strategies have combined multimedia campaigns and technology with community programs at schools or family doctors’ offices [36].

### Rationale

The Back to Reality Series is an innovation using a medium that appeals to a traditionally more challenging cohort to engage in treatment. By simulating the experience of psychosis, the Back to Reality Series is expected to improve appreciation of the impact of cannabis use on psychosis. To date, the Back to Reality Series is the only set of video games in Canada or internationally that addresses cannabis use, pathways to care, and the risk of psychosis, inspired by data derived from studies involving people of Black African and Caribbean descent with a first episode of psychosis [37-39]. This project aims to evaluate the feasibility of the Back to Reality Series as a knowledge translation product for young people experiencing a first episode of psychosis, particularly those from Black communities. This knowledge translation approach aims to effectively disseminate the research knowledge to those who might benefit from understanding the risks.

This study will reveal how racial identity, gender, psychosis, and cannabis use intersect and uniquely impact the lives of young people with a first episode of psychosis and cannabis use disorder from Black racialized communities. It will involve a series of qualitative interviews and conversations with participants about cannabis and psychosis, digging deeper into their experiences to understand how these episodes shaped them. We explore what sets young people of Black African and Caribbean descent apart from other ethnicities regarding their cannabis use.

### Study Aims

#### Perspectives on Cannabis and Psychosis

This study will examine the perceptions of the mental health effects of cannabis on psychosis based on young people’s perspectives among Black African and Caribbean first- or second-generation immigrants with a first episode of psychosis and a cannabis use disorder. These perceptions will be explored before and after the use of the Back to Reality Series. To this end, the study will examine narratives, opinions, and personal experiences to elucidate how the relationship between cannabis and psychosis is processed to appreciate why they think the way they do. The study will outline the participants’ understanding of the emotional, social, and mental health impacts of ongoing cannabis use on the first episode of psychosis. We will explore stories about the perceptions of the benefits and harms of cannabis, applying a race and gender lens to glean perspectives before and after playing the Back to Reality Series.

#### Insight and Knowledge Acquisition

The second objective is to establish the feasibility of using the Back to Reality Series to translate knowledge about the mental health effects of cannabis. Knowledge acquisition will be measured by comparing scores on a quiz after playing the Back to Reality Series versus a control game. After playing whichever game remains, the participants’ perceptions of the effects of cannabis use on psychosis will be explored to converge qualitative data before and after playing the Back to Reality Series.

#### Development of a Strong Participatory and Community Engagement Component

The third objective is to develop a strong participatory and community engagement component based on the inclusion of an advisory council. The proposal builds on co-creation and capacity building, which increases the project’s community relevance.

## Methods

### Design

This mixed methods study aims to conceptualize what young people with early phase psychosis/cannabis use disorder understand about the relationship between cannabis and psychosis, focusing on people from racialized backgrounds. All aspects of the research will be conducted virtually. Participants will be interviewed over Zoom, and the interviews will be video and audio recorded to observe emotional responses and to produce verbatim transcripts for qualitative analysis. Consent will be obtained over the Zoom platform. The research staff consisting of research students, research assistants, and a research coordinator will be invited to self-identify as one of the designated underrepresented groups in their cover letter (Black or person with lived experiences of psychosis and cannabis use). This process will help to increase concordance between researchers and participants, and foster safe spaces for dialogue around complex issues such as race and cannabis use [40]. The principal investigator will train the research staff. An advisory council will help create a knowledge dissemination plan involving the Back to Reality Series to generate ideas about promoting it to early psychosis intervention services and Black racialized communities. A French-language version of the Back to Reality Series is currently being developed, and 10 French-language participants will be recruited for this small-scale demonstration project once completed. The details of this protocol will be published elsewhere once the games have been translated into French.

### Ethics Approval

Ethics approval has been obtained from the Hamilton Integrated Research Ethics Board (number 13417) and the Western Research Health Sciences Research Ethics Board in London, Ontario. Full approval was granted May 27, 2021 until April 20, 2022. On April 9, 2022 received an annual approval extension from April 20, 2022 to April 20, 2023. We are awaiting approval from the Toronto Academic Health Sciences Network, the Ottawa Health Science Network Research Ethics Board, and the Research Ethics Board of the CIUSSS de l’Ouest-de-l’île-de-Montréal.

### Community Engagement

The advisory council will involve Black community members, clinicians, family members, Black youth, and young people with lived experiences. It will include engagement of the following community partners: young people from the Free for All Foundation, a charitable organization serving families and youth from Black communities in Brampton, Ontario; the Institute for the Advancement of Mental Health (formerly the Schizophrenia Society of Ontario), serving people with serious mental illness and their family members; and the Early Psychosis Intervention in Ontario Network, which involves over 50 early psychosis intervention clinical programs throughout the province of Ontario. The members will not be participants in the study. It is not a steering committee because the advisory council does not manage the operations of the research, manage the research progress, or conduct the research. The advisory council members advise the researchers about the research priorities and interpretations about the participants’ perspectives on cannabis and psychosis, as well as their insights and knowledge acquisition after playing the game. The advisory council will engage in quarterly knowledge exchange meetings to (1) help develop a plan to disseminate the Back to Reality Series for young people, particularly youth from Black racialized communities, (2) reflect upon and review educational content inspired by the video games, (3) monitor and review the representation of psychosis and Black or racialized persons within the research findings, (4) give feedback about qualitative analysis of the findings, and (5) help integrate the Back to Reality Series into early intervention care or use by community agencies. 

To create an environment where community members and people with lived experiences feel supported in participating in the research process, the advisory council will adopt the guidelines promoted by the Strategy for Patient-Oriented Research of the Canadian Institute for Health Research [41] as follows: mutual respect, clarification of roles, co-creation of knowledge, support and build on equity, diversity, and inclusion principles.

### The Back to Reality Series and the Control Game Morpheus’ Spell

**Figure 1:**
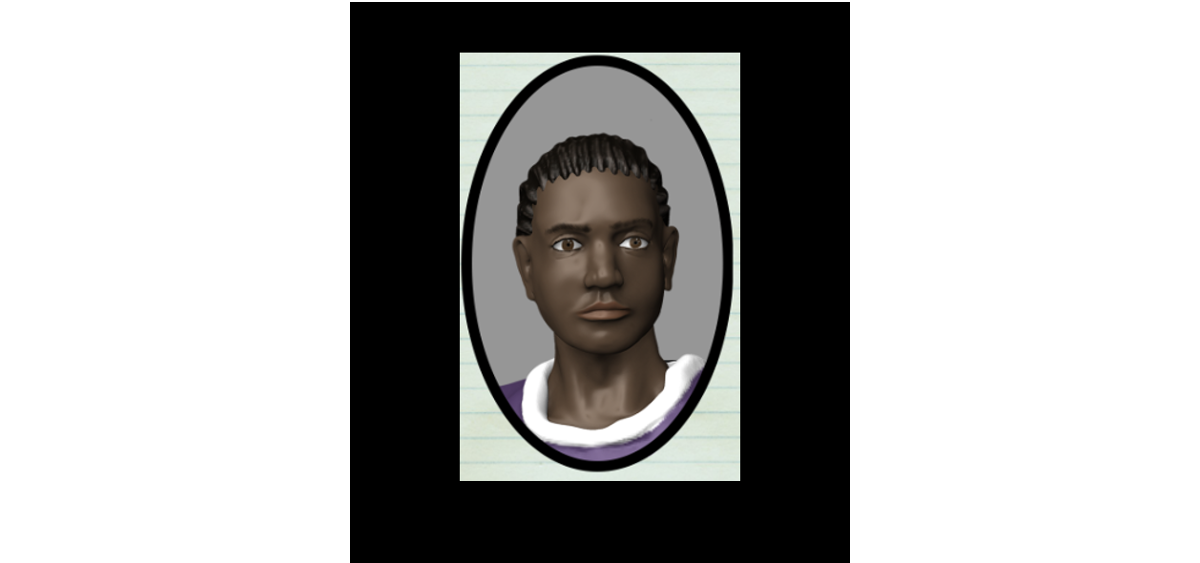
The Back to Reality Series character Harry.

The Back to Reality Series is a set of video games designed to increase knowledge about cannabis risks for underage youth and young people experiencing a first episode of psychosis. The Back to Reality Series consists of the following 3 video games: Harry’s Journey, Harry’s Journal, and Harry’s PathwaysToCare Map (Figure 1). Harry’s Journey tells the story of Harry (a teenager in his senior year of high school; second-generation Jamaican descent) who starts to experience psychosis after using cannabis regularly for 4 years. Harry’s Journal takes scenes from Harry’s Journey to illustrate psychiatric symptoms associated with psychosis. The PathwaysToCare Map displays 3D replicas of youth mental health and addiction services to help players navigate the mental health care system. It is novel because it uses gaming technology to portray psychosis and because the interactivity allows the players to explore the potential harms and benefits of cannabis use. 

Archie produced the games adopting serious game design methodology [27] with an integrated knowledge translation community involving Black youth, young people with lived experiences of cannabis and psychosis, family members, game designers, clinicians, and researchers [42]. Focus group feedback from family physicians revealed that the Back to Reality Series delivered content relevant for youth in their practice about mental health and addiction [43]. Family physicians recommended offering the games to youth while patients were waiting for their appointments.

There have been small demonstration projects to pilot the use of the Back to Reality Series. Ten participants between 17 to 30 years of age with a first episode of psychosis felt that Harry’s Journey realistically portrayed psychosis experiences, and they enjoyed playing it [44]. Fifty-five youth experiencing homelessness (16-19 years of age) were randomized to play the Back to Reality Series versus a control game. Participants playing the Back to Reality Series demonstrated a significant mean knowledge test score advantage (mean 6.8, SD 1.6) compared to those playing the control game (mean 5.5, SD 1.9; *P*=.02) [45]. In 2020, 10 undergraduate students created tutorial objectives and content inspired by the Back to Reality Series. Using the Back to Reality Series as the focus of dialogue, student-led tutorials were conducted involving 9 Black youth (16-19 years of age) as participants. The participants demonstrated significant increases in their knowledge about cannabis, mental health, and pathways to care after playing the Back to Reality Series and engaging in the tutorials [42]. Qualitative analysis of the feedback revealed that the content was meaningful to them (eg “I guess the fact that Harry lived in a realistic teenage lifestyle that like a lot of youth can relate to and that made it more meaningful cause it felt more relevant.”) [42].

The control game “Morpheus’ Spell” is inert. It is a spelling “pop culture” digital game with no mental health content. It failed to increase knowledge about mental health and addiction when undergraduate students were randomized to play the Back to Reality Series first, obtaining a Psychosis and Cannabis Test (PCT) Quiz score of 7.55 (SD 1.04) versus a score of 7.82 (SD 0.40; *P*=.28) after playing the Back to Reality Series followed by Morpheus’ Spell [46].

### Visit 1: Baseline Assessments

#### Study Population

The eligibility criteria are as follows: (1) meet the criteria for a first episode of psychosis defined as the first illness episode involving psychosis symptoms; (2) meet DSM-5 (Diagnostic and Statistical Manual of Mental Disorders, 5th Edition) criteria for a psychotic disorder and a lifetime or current cannabis use disorder (up to 45% of people with a first episode of psychosis will meet the criteria for cannabis use disorder [16,47,48]; patients diagnosed with cannabis-induced psychosis by definition meet the criteria for both conditions [49] and will be eligible); (3) be between the ages of 16 and 30 years; (4) self-identify as a Black African or Black Caribbean first- or second-generation immigrant (English speaking), or belong to any ethnic group; (5) be a client of an early psychosis intervention with up to 12 months of service; and (6) be fluent in English.

Participants will self-identify and be divided into the following disaggregated ethnic and racialized groups: Black African (n=24), Black Caribbean (n=24), and any ethnicity (n=24). There may be significant differences in social and community norms for cannabis use between Black African and Caribbean groups and differences in English proficiency, educational attainment, and immigrant status. Sex will be defined based on sex assignment at birth. Gender will be self-assigned as man, woman, transgender, fluid, two-spirit, and non-binary. Equal numbers of men (including transmen) and women (including transwomen) will be enrolled for each racialized group. Participants will self-identify as nonimmigrant, or first- or second-generation immigrant.

#### Recruitment of Study Participants

A convenience sample will be recruited. The following 5 early psychosis intervention sites will recruit the participants: London, Toronto, Hamilton, Ottawa, and Montreal. All early psychosis intervention sites share the same entrance criteria for admission and offer similar services. The sites provide a pool of over 1000 patients with a first episode of psychosis. Based on prior early intervention studies involving this population, at least 30% will be of Black African and Caribbean descent [37,39]. Staff at each site will receive training about recruitment, approach eligible clients, and obtain consent for the research coordinator to contact them. Participants can access laptops on loan from the study or, when pandemic restrictions allow, can access computers at early intervention programs.

#### Data Collection

**Figure 2:**
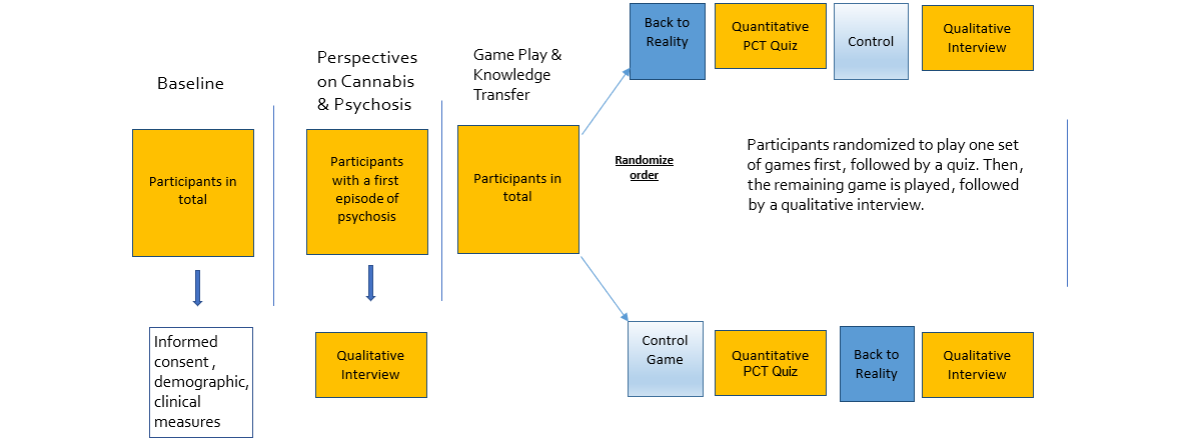
Study design. PCT: Psychosis and Cannabis Test.

This phase of the project involves baseline clinical assessments. Research staff will conduct an individual interview to obtain baseline demographic/clinical measures and illicit narratives exploring the perceptions of the effects of cannabis use on psychosis (Figure 2).

#### Assessment

Demographic data will be collected on gender, sex, age, ethnicity, education, living circumstances, language, income, immigrant status (first or second generation), employment, disability status, and DSM-5 diagnoses.

#### Measures

##### Clinical and Sociodemographic Measures

The Diagnostic Assessment Research Tool (DART) for DSM-5 diagnoses [50] will be used as an interview guide to help establish the diagnosis. Baseline measures will also be drawn from demographic tools used in the Ontario Community Mental Health Evaluation Initiative [51]. This tool collects information on marital status, ethnicity, employment status, immigration status, and other sociodemographic characteristics.

##### Drug Use and Abuse

The prevalence rates of cannabis and alcohol use disorders will be assessed using the DART for lifetime and the 12-month period prior to the baseline assessments. The DART is an open-access semistructured interview developed by researchers at McMaster’s Department of Psychiatry and Behavioral Neurosciences for clinical and research assessments of DSM-5 diagnoses for mental disorders. The Schizophrenia and Substance Use Disorders modules will be adopted for this project [50].

##### Drug Abuse Screening Tool

The Drug Abuse Screening Tool (DAST) will be used to track cannabis use indicative of abuse before clinic registration and at 12 months. The DAST monitors drug use problems during the previous 12-month period. The DAST identifies drug abuse when a score of 6 or more is obtained. The lowest score is 0, and the highest score is 20 [52]. It has been used in previous studies to identify substance use disorder among young people with a first episode of psychosis [16].

##### Insight

The Birchwood Insight Scale will be completed. It contains items about illness awareness, symptom recognition, and the need for treatment. It has been validated in schizophrenia and patients with a first episode of psychosis [53].

The Cannabis Experience Questionnaire has been used to measure the intoxication effects of cannabis among cannabis users and has been validated with confirmatory factor analysis [54]. It has been used in early phase psychosis to track the frequency and THC content of cannabis use [55]. It assesses the frequency of use (every day, more than once a week, about once a week, about once a month, a few times each year, only once or twice, and never) [55]. It has been used in epidemiological studies involving first-episode psychosis participants in the United Kingdom to estimate the type of cannabis used. This aspect was developed by collecting data on the THC content of cannabis confiscated by police [56]. We have adapted it for the Ontario context by estimating THC content based on a Marketplace survey of products sold at dispensaries in Canada [57,58]. It includes the following question: What potency of cannabis do you/did you usually buy? (Indicate the most frequent if this varies). The response options are (1) Low potency, 5% or less (THC content labeled or hash oil); (2) Medium potency, 6%-14% (THC content labeled at a government-sponsored dispensary); (3) High potency, 15%-29% (most dispensaries not licensed by the government); (4) Extremely high potency, 30%-100% (synthetics, concentrates, shatter, dab, or spice). 

#### Analyses

##### Quantitative Analysis

Descriptive statistics (mean and standard deviation for normally distributed continuous variables, median for variables with skewed distribution, and frequency and percentage for categorical variables) will be used to depict the demographic and clinical characteristics and will explore the relationship between the variables of interest. Relevant tests of significance, including chi-square, correlation, and regression tests, will be performed.

### Visit 2: Perspectives on Cannabis and Psychosis

#### Research Questions

The primary research questions about the perceptions of cannabis and psychosis are as follows: How do young people with a first episode of psychosis and cannabis use disorder from Black racialized communities conceptualize the mental health impacts of cannabis on psychosis? How do gender and ethnicity influence the messages?

#### Study Population and Recruitment

See Visit 1: Baseline Assessments for criteria and procedures. The same participants will take part in this phase of the study.

#### Data Collection

##### Qualitative Interview

The research staff will ask participants to share their stories and perspectives involving cannabis and psychosis. Semistructured open-ended questions will be used to elicit narratives on mental health and cannabis use experiences. Stories will also be elicited about the age of first use, frequency of use, potency of products, choice of products, route of administration, and views on how gender or ethnicity influences cannabis use.

The interviews will be recorded and transcribed verbatim. The Sex- and Gender-Based Analysis Plus will be used to ensure the data are collected and analyzed in a manner sensitive to gender (sociocultural) and to integrate factors, such as race, language, age, immigration, or disability [59]. The research staff will explore within-group themes that are contextually grounded in the following: (1) race and ethnicity as social constructs (for example, Black African versus Black Caribbean), (2) gender (men/transmen versus women/transwomen versus fluid/nonbinary/two-spirit), (3) immigration status (first or second generation), and (4) study site (for example, Montreal versus London to capture variations based on geography).

#### Qualitative Analysis

The data will be thematically analyzed as described by Braun and Clark [60]. The research staff will conduct thematic analysis, code and label data extracts, and search for phrases and imagery related to cannabis and psychosis, focusing on different perspectives based on gender and ethnicity. This analysis will be presented to the advisory council to help formulate meaning from the data and to help create visual models of the constructs.

#### Sample Size

Twelve or more participants from each of the 2 ethnic groups and the 2 largest gender groups should be sufficient to reach saturation of the perspectives for each group by the third visit. Saturation has been shown to occur within the first 12 interviews within a group [61].

### Visit 3: Gameplay and Knowledge Transfer

#### Research Questions

The research questions are as follows: (1) How do the themes about cannabis and psychosis change after playing the Back to Reality Series? (2) What is the game’s impact on knowledge acquisition about cannabis and psychosis?

#### Hypothesis

We hypothesize a significantly greater knowledge test score (>18%) in the group first exposed to the Back to Reality Series compared to the control game. We expect this finding for Black African and Black Caribbean groups, Black men, Black women, and all ethnic groups with a first episode of psychosis and cannabis use disorder.

#### Study Population and Recruitment

See Visit 1: Baseline Assessments for criteria and procedures. The same participants will take part in this phase of the study.

#### Study Design

This study phase involves quantitative and qualitative components to establish the feasibility of knowledge acquisition through gameplay involving the Back to Reality Series. Knowledge acquisition will be measured using scores on a quiz, and changes in insight will be gleaned from qualitative inquiry into changes in perceptions about cannabis and psychosis before and after playing the game.

#### Data Collection and Procedures

The video games will be streamed over Zoom by the research staff. Participants will be randomized to determine the gameplay order (either the Back to Reality Series or the control game will be played first). A software program embedded within the download of the Back to Reality Series randomly assigns the order in which each participant plays the first game on a 1:1 basis. The randomization is not to allocate participants into groups. The experimental and control games are packaged on the screen to protect the blinding at the outset. However, the video games are not associated with preconceived notions of therapeutic benefit, which minimizes bias. The video games are password protected, so participants will not have had prior access.

Subsequently, participants will complete a quiz measuring factual knowledge about the impact of cannabis on psychosis. Participants are randomized so that differences in quiz scores can be attributed to gameplay interventions, which is an advantage over pre-post gameplay designs for quantitative analysis. This process also avoids having participants complete the same or similar quiz twice in a day. The research staff will conduct the knowledge test by displaying the questions to the participants and reading out each item. After completing the quiz, participants will immediately play whichever game remains. Thus, if the participant was randomized to play the Back to Reality Series first, the control game, Morpheus’ Spell, will subsequently be played.** **Each participant will complete a gameplay survey about the Back to Reality Series. Finally, participants will undergo a second qualitative interview to discuss ideas and impressions from their gameplay experiences and reflect upon their views about the relationship between cannabis and psychosis after playing the game.

#### Measures

##### PCT

The PCT Quiz assesses relevant knowledge about the relationship between cannabis and psychosis. It takes about 10 minutes to complete and involves 10 multiple-choice questions worth 1-point each, with scores ranging from 0 to 10. The researchers will read the questions aloud to reduce the impact of reading literacy. The details are described elsewhere regarding the development of the quiz, and its reliability and validity [42].

##### Postgameplay Survey

This survey explores the satisfaction with the gameplay experience. It was adapted from a randomized control study of video games designed to increase knowledge about antenatal care [62]. The items include “comfort with gameplay,” as well as enjoyment of the game, graphics, music, and story. Each item is rated as “Yes” (1 point) or “No” (0 points). Additional questions include the frequency of video gameplay and the types of devices used to play video games. The survey has been piloted with the Back to Reality Series involving Black youth [42].

#### Data Collection

##### Gameplay, Quantitative Test, and Qualitative Inquiry

This section will take 60-120 minutes. Each participant will be provided with a link to download the set of video games. A software program will randomly assign the order in which each participant plays the first game, the Back to Reality Series, or the control game on a 1:1 basis. It takes 45 minutes in total to play all the video games. Subsequently, the research staff will conduct the PCT Quiz by displaying the questions to the participants and reading out each item. Finally, the participants will play whichever game they had not played first.

##### Qualitative Interview

After a short break, the research staff will conduct qualitative interviews about the gameplay experiences and the perceptions of the mental health effects of cannabis and psychosis after playing the game. The participants will share how the perceptions of their own personal experiences with psychosis and cannabis might be influenced by their gameplay experiences. Participants will be asked about their views on the main character’s use of cannabis and their opinions about using video games for youth from Black communities.

#### Analyses

##### Qualitative Analysis

The same qualitative analysis and sample size procedures as outlined in Visit 2: Perspectives on Cannabis and Psychosis will be adopted for this analysis. However, the data sets from *Perspectives on Cannabis and Psychosis* and *Insight and Knowledge Acquisition* will be integrated during the analysis to detect changes in perception comparing pregame and postgame data sets to identify changes in insight or opinions. Triangulation of the feedback from different disaggregated racialized and gender groups will be performed to compare and contrast the views on cannabis and psychosis in order to identify relevant themes and ideas [63].

##### Quantitative Analysis

We will compare the performance of the groups on the PCT Quiz using the *t* test. A minimum difference of 18% in PCT Quiz scores represents a statistically and meaningfully significant increase in knowledge, based on a pilot study [45].

#### Sample Size

Based on pilot data involving 10 participants with a first episode of psychosis, the mean baseline score was 6.5 (SD 1.3). The effect size is 1.17 (18% × 6.5) to detect a difference of 18%. The standardized effect size (E/S) is 0.9 (1.17/1.3 [effect size/SD]). The power calculations [64] suggest that 20 participants are sufficient per group. With a minimum of 24 participants per gender and racialized group at visit 1, there would be at least 20 participants per group by visit 3 (up to 4 weeks later), with a projected dropout rate of 15%.

## Results

We began recruiting participants for this study in September 2021. So far, we have had 2 advisory committee meetings, one in October 2021 and the second in December 2021. Recruitment is being tailored to fit with the pandemic-related restrictions. The recruitment target is expected to be met by December 2022. Full analysis of the results will be completed subsequently.

## Discussion

The anticipated findings for this study center around knowledge exchange about cannabis and psychosis, and engaging young people experiencing a first episode and, in particular, those of Black African and Caribbean descent. We expect the video games to encourage meaningful discussions about cannabis and psychosis. We hope the characters, narrative, interactivity, and video gameplay promote retention and engagement. The PCT Quiz will verify knowledge acquisition. Attitudes and satisfaction will be documented by qualitatively analyzing themes reviewed by the advisory council. The Back to Reality Series, combined with tutorials led by university students, has been shown in a pilot study involving healthy participants to be enjoyable and to transfer knowledge about cannabis and psychosis to youth from Black communities [42].

The strength of this protocol includes community engagement provided by the advisory council, which promotes research priorities and interpretations that are more acceptable to youth with serious mental illness and young people from Black communities. A limitation of the design includes the lack of follow-up to document the long-term impact of the games.

Few Canadian studies report cannabis use based on race or ethnic groups; nevertheless, ethnic differences have been found between young people of Black African background and those of Black Caribbean background, with the latter group more likely to report problematic use [23]. Studies conducted in the United States involving healthy populations have shown that Black youth are more likely than White youth to report self-medicating with cannabis to relieve physiological symptoms associated with anxiety [65]. However, many studies suggest that youth use cannabis because they believe it will alleviate anxiety symptoms regardless of race, but no studies to date indicate that youth self-medicate to ease psychosis symptoms [66,67]. This study is unique because it will explore the intersections of race, ethnicity, and cannabis use among young people experiencing their first episode of psychosis.

Future directions include using digital video games as psychoeducational products that explore credible scientific knowledge about the risks and benefits of cannabis use. The video games provide a unique opportunity to reach young people from Black communities with the help of community partners. The Back to Reality Series could offer a coherent knowledge transfer strategy for education and engagement. Moreover, the Back to Reality Series could become a sustainable evidence-based online knowledge translation product, reaching an appropriate audience through networks connected to early interventions in psychosis and community agencies. Validation of the psychoeducational value of the Back to Reality Series could support future studies examining its ability to promote readiness for change and its ability to improve not only insight but also engagement with cannabis use disorder treatments offered by early intervention programs.

## Appendix

Multimedia Appendix 1 CIHR peer review.

## Abbreviations

CBT: cognitive behavioral therapy
DART: Diagnostic Assessment Research Tool
DAST: Drug Abuse Screening Tool
DSM-5: Diagnostic and Statistical Manual of Mental Disorders, 5th Edition
PCT: Psychosis and Cannabis Test
THC: delta-9-tetrahydrocannabinol
