# Supplementary material for: Insights About Cannabis and Psychosis Using Video Games for Young People With a First Episode of Psychosis, Particularly Those From Black Racialized Communities: Protocol for a Mixed Methods Study
Source: JMIR Res Protoc. 2022 May 20;11(5):e36758. doi: 10.2196/36758 (PMC9166641; doi:10.2196/36758)
Supplement: Multimedia Appendix 1 [file resprot_v11i5e36758_app1.pdf]

March 12, 2021 / 12 mars 2021

Canadian Institutes of Health Research / Instituts de recherche en santé du Canada

Notice of Decision / Avis de décision

Application Number/Numéro de la demande:	454732
Committee Code/Code du comité:	CNB

Applicants/Candidats:	Archie, Suzanne<br>Langill, Gordon<br>Serravalle, Michael
With/Avec:	Anderson, Kelly K; Ayonrinde, Oyedeji; Baines, Alexandra S; Bardell, Andrea; Cheng, Chiachen; Ferrari, Manuela; Grant, Christina N; Johnson, Natasha I; Kozloff, Nicole; Olagunju, Andrew T; Palaniyappan, Lena K; Sadeh, Elham
Institution paid / Établissement payé:	McMaster University
Title/Titre :	Insights about Cannabis and Psychosis: How do young people with early psychosis conceptualize the link between cannabis and psychosis, particularly those from Black racialized backgrounds?

**Competition Outcome/Résultats du concours:**	Catalyst Grant : Cannabis and Mental Health / Subvention catalyseur : Cannabis et santé mentale
**Number in competition/ Nbre de demandes dans le concours :**	**41**
**Number approved/ Nbre de demandes approuvées:**	18

**Decision on your application/ Décision sur votre demande:**	**Approved/Approuvée**
**Average annual amount/ Montant annuel moyen:**	$115,000
**Term/Durée:**	**1 Year(s), 0 Month(s)**

Peer Review Committee Recommendation, for your information and use/
Recommandation du comité d'examen par les pairs, pour fins d'information et d'utilisation :

Peer Review Committee Recommendation, for your information and use/
Recommandation du comité d'examen par les pairs, pour fins d'information et d'utilisation:

Pool/Classe:	Cannabis use and early phase psychosis / Consommation de cannabis et stade initial de la psychose
Number reviewed in that Pool / Nbre de demandes examinées dans cette classe:	6
Number approved in that Pool / Nbre de demandes approuvées dans cette classe:	1
Application rank within the Pool / Rang de la demande dans cette classe:	1
Rating/ Cote:	4.15
Additional Funding Opportunities / Opportunités de financement additionnelles:	Cannabis use and psychosis in adult populations / Consommation de cannabis et psychose dans les populations adultes
Decision on Additional Funding Opporunities / Décision sur les oppotunités de financement additionnelles:	Not Approved/Non Approuvée

*** Applications receiving an overall rating of less than 3.5 will not be considered for funding. / Les demandes qui ont reçu une cote inférieure à 3.5 ne sont pas admissibles.

This document is for information only. Official payment is stated on the CIHR Authorization for Funding.
Document à titre d'information seulement. Le paiement officiel est indiqué sur l'autorisation de financement des IRSC.

Canadian Institutes of Health Research
160 Elgin Street, 9th Floor
Address Locator 4809A
Ottawa, Ontario K1A 0W9

Instituts de recherche en santé du Canada
160, rue Elgin, 9e étage
Indice de l'adresse 4809A
Ottawa (Ontario) K1A 0W9

F. Peer Reviewed Grants Level 1 Grants, 13a. Principal Investigator $115K

March 12, 2020

Institute of Aging
Institute of Cancer Research
Institute of Circulatory and Respiratory Health
Institute of Gender and Health
Institute of Genetics
Institute of Health Services and Policy Research
Institute of Human Development, Child and Youth Health
Institute of Indigenous Peoples' Health
Institute of Infection and Immunity
Institute of Musculoskeletal Health and Arthritis
Institute of Neurosciences, Mental Health and Addiction
Institute of Nutrition, Metabolism and Diabetes
Institute of Population and Public Health

Institut du vieillissement
Institut du cancer
Institut de la sante circulatoire et respiratoire
Institut de la santé des femmes et des hommes
Institut de génétique
Institut des services et des politiques de la santé
Institut du développement et de la santé des enfants et des adolescents
Institut de la santé des Autochtones
Institut des maladies infectieuses et immunitaires
Institut de l'appareil locomoteur et de l'arthrite
Institut des neurosciences, de la santé mentale et des toxicomanies
Institut de la nutrition, du métabolisme et du diabète
Institut de la santé publique et des populations

Doctor Suzanne Archie
Department of Psychiatry & Behavioural Neuroscience
McMaster University
St. Joseph's Healthcare Hamilton
East Region Mental Health Clinic
2757 King Street East
Hamilton, ON L8G 5E4

Dear Dr. Archie,

On behalf of the Canadian Institutes of Health Research (CIHR) and the Mental Health Commission of Canada (MHCC), we are pleased to inform you that your recent application submitted to Catalyst Grant: Cannabis and Mental Health competition entitled, "Insights about Cannabis and Psychosis: How do young people with early psychosis conceptualize the link between cannabis and psychosis, particularly those from Black racialized backgrounds" has been approved for funding.

The review documents for your application are available online through ResearchNet at: www.researchnet-recherchenet.ca.

MHCC will contact you directly regarding the process to execute the grant agreement.

As CIHR does not notify co-applicants of the decision, we ask that you inform those individuals involved, along with their research institutions (if different from your own), of the outcome of this application.

Should you require additional information on the review process, please contact the CIHR Contact Centre at support-soutien@cihr-irsc.gc.ca. Please do not contact the officers or members of the peer review committee.

Congratulations on your success in this competition.

Sincerely,

Bryan Lemire

Bryan Lemire, MPA
Acting Manager, Program Design and Delivery
Research Programs Portfolio

Review Type/Type d'évaluation: Committee Member 1/Membre de comité 1
Name of Applicant/Nom du chercheur: Archie, Suzanne
Application No./Numéro de demande: 454732
Agency/Agence: CIHR/IRSC
Competition/Concours: 2020-11-19 Catalyst Grant: Cannabis and Mental Health/Subvention catalyseur : Cannabis et santé mentale
Committee/Comité: Catalyst Grant: Cannabis and Mental Health/Subvention de catalyseur: Cannabis et santé mentale
Title/Titre: Insights about Cannabis and Psychosis: How do young people with early psychosis conceptualize the link between cannabis and psychosis, particularly those from Black racialized backgrounds?

Assessment/Évaluation:

- The study aims to evaluate the use of a new video game (SERIES) focusing on black youth as a hero with psychosis. To be included in the study, a participant needs to have had a first episode of psychotic disorder and a cannabis use disorder. The standard clinical interview for DSM-V will be used to diagnose participants to be included in the study.
- Three sets of data will be collected; a) qualitative interview before the game, quantitative interview- which is meant to explore experiences and perspectives of cannabis and psychosis b) quantitative test using PCT quiz, a validated tool to measure the knowledge change on the connection between cannabis and psychosis and; c) qualitative interview focusing on experiences playing the game.
- It is not clear why the researchers opted for a pre-game qualitative interview instead of a pre-game quantitative quiz, given that they intend to use a t-test to measure the change in knowledge (pre and post-intervention change).
- The explicit role that the advisory council will play to respond to research objective three- i.e., to develop a strong participatory and community engagement component is not fully developed.
- The research is well supported by the community based on the letters of support from the partner agencies
- There is a strong mentorship component judging by the students both in graduate and undergraduate program involved in the study
- This research builds on previous work with the community that led to the development and validation of the measurement tools and the video series.
- The PI and her team have a track record of engaging in research with the community.
- The team is well-positioned to conduct the study
- The budget is justified well.

Review Type/Type d'évaluation: Committee Member 2/Membre de comité 2
Name of Applicant/Nom du chercheur: Archie, Suzanne
Application No./Numéro de demande: 454732
Agency/Agence: CIHR/IRSC
Competition/Concours: 2020-11-19 Catalyst Grant: Cannabis and Mental Health/Subvention catalyseur : Cannabis et santé mentale
Committee/Comité: Catalyst Grant: Cannabis and Mental Health/Subvention de catalyseur: Cannabis et santé mentale
Title/Titre: Insights about Cannabis and Psychosis: How do young people with early psychosis conceptualize the link between cannabis and psychosis, particularly those from Black racialized backgrounds?

Assessment/Évaluation:

1. Research Approach

The objectives of this project is to (1) examine perceptions of the mental health effects of cannabis on psychosis; (2) Establish the feasibility of knowledge acquisition [about mental health effects of cannabis on psychosis] comparing the SERIES versus a control game; and (3) Develop a strong participatory and community engagement component. SERIES is a KT product in the form of a videogame that can depict psychosis-like experiences, and that can address cannabis use. In terms of rationale, the authors note there is no targeted intervention for cannabis use disorder among Black immigrants with psychosis, the perceived effects of cannabis on psychosis not understood within this group, and use of a videogame intervention can be effective. SERIES has been validated with homeless youth, including Black youth.

A mixed methods study will be used and conducted virtually. Youth will be Black immigrants experiencing psychosis (new cases) and CUD. They will collect quant/qual baseline data – this is well described. The next visit will give youth a link to the videogames and collect data related to key outcomes. These procedures are well described, and well thought out. There will then be another qualitative interview. Figure 2 was helpful to visualize the key components of the study and their timing. The advisory council they will create is also well described and clearly well thought out. The team has organized and provided the questions they will use in the interviews, and it is clear that much work has been done to plan and get ready to launch the study.

2. Applicant(s)

The PI is Co-Chair of the Anti-Black Racism Task Force Department of Psychiatry and Behavioural Neurosciences at McMaster University and Member Peter Boris Centre for Addictions Research. The PI was past Clinical Director of the Cleghorn Early Psychosis Intervention Clinic at St Joseph's Hospital from 2004 to 2019. She was a scientific advisor to the Schizophrenia Society of Canada. She holds another gran on the topic under study in this proposal (2020-2022) for $25,000, and has been a co-applicant or Co-I on a number of other recent grants on these topics. Her publication record is modest with 11 publications between 2015-2020, and only a few as first or last author. The team includes Co-Is, collaborators and a knowledge user that are experienced in the field, interdisciplinary and strong.

3. **Impact of the Research**

The team seeks to find out how racial identity, gender, psychosis and CUD intersect to impact the lives of youth people. Based on my review, I agree that the team has put together a project that can achieve this aim.

3. Open Science and Knowledge Translation Plan

The KT plan is strong and detailed.

Review Type/Type d'évaluation: Committee Member 3/Membre de comité 3
Name of Applicant/Nom du chercheur: Archie, Suzanne
Application No./Numéro de demande: 454732
Agency/Agence: CIHR/IRSC
Competition/Concours: 2020-11-19 Catalyst Grant: Cannabis and Mental Health/Subvention catalyseur : Cannabis et santé mentale
Committee/Comité: Catalyst Grant: Cannabis and Mental Health/Subvention de catalyseur: Cannabis et santé mentale
Title/Titre: Insights about Cannabis and Psychosis: How do young people with early psychosis conceptualize the link between cannabis and psychosis, particularly those from Black racialized backgrounds?

Assessment/Évaluation:

Research Approach

The proposed application falls within the scope of the CIHR Catalyst Grant: Cannabis and Mental Health to generate lived experience-based knowledge on potential benefits/harms of cannabis use and mental health implications, support community-based/population-specific research, facilitate the development of new evidence to inform policies, and connect existing knowledge translation (KT) efforts across Canada.
The overall goal of this mixed-methods project is to explore perceptions about cannabis and psychosis from Black and racialized people, develop KT products to compare before and after exposure, and foster community engagement of stakeholders to create a referral strategy to boost young people from Black and racialized communities.

The literature review is comprehensive, timely, and relevant to the proposed research design, which is thorough and appropriate. This research will be guided by an Advisory Council of Black and Racialized peoples with lived experiences that ensure relevance and the cultural appropriateness of the study. However, it is not clear, how all research participants will have access to the virtual platform given their diverse socio-economic backgrounds. It is also not clear, how the data collection and knowledge translation processes will address and mitigate the potential barriers if the COVID-19 pandemic continues to prevail.

The proposed project considers SGBA+ to ensure that data collection and analysis are sensitive to gender and identify factors such as race, language, age or disability. As the proposal describes, specific consideration will be given to self-assigned identify factors (such as man, woman, transgender, fluid, two-spirit). Equal numbers of men (including transmen) and women (including transwomen) will be enrolled for each racialized group).

The proposal builds on strong participatory research, co-creation concept, capacity building, and community engagement components, which increases the proejct’s community relevance.

Applicants:
The applicants form a multidisciplinary team of researchers and collaborators and well-positioned to undertake this research. The research team including NPI, co-applicants, knowledge users, and collaborators has several years of experience, expertise, and proven track record in the area of mental health and addiction research and related community-based work. The NPI has significant experience in the field of addiction and mental health research with successful research funding history, peer-reviewed publications (n=13), presentations (n=4), and knowledge translation and mentorship experiences.

The participants received strong letters of support or collaboration from a range of stakeholders that demonstrate their significant contribution to this field of research.

**Review Type/Type d'évaluation:**	Committee Member 3/Membre de comité 3
**Name of Applicant/Nom du chercheur:**	Archie, Suzanne
**Application No./Numéro de demande:**	454732
**Agency/Agence:**	CIHR/IRSC
**Competition/Concours:**	2020-11-19 Catalyst Grant: Cannabis and Mental Health/Subvention catalyseur : Cannabis et santé mentale
**Committee/Comité:**	Catalyst Grant: Cannabis and Mental Health/Subvention de catalyseur: Cannabis et santé mentale
**Title/Titre:**	Insights about Cannabis and Psychosis: How do young people with early psychosis conceptualize the link between cannabis and psychosis, particularly those from Black racialized backgrounds?

Assessment/Évaluation:

Impact of Research: The proposed study will be the first of its kind in Canada to reveal how racial identity, gender, psychosis, and cannabis use intersect and uniquely impact the lives of young people with FEP and CUD from Black racialized communities. This project intends to give voice to the people from racialized Black and other communities to generate new knowledge about the meaning of race, cannabis use, and psychosis to inform a new KT product to address knowledge gaps about early phases of psychosis from cannabis use. The emerging KT product will likely provide new recruitment strategies that EPI clinicians could use to engage more young people from marginalized communities into care.

Open Science & Knowledge Translation Plan:
The study's research protocol will be published in an Open Access journal. Additionally, the research findings will be presented at conferences, workshops, and online. The research project aims to share co-developed knowledge via monthly sharing circles and co-production of KT products in the end.

Budget: the funding ask seems appropriate. Surprisingly, the project proposal has no funding allocation for KT activities.

Review Type/Type d'évaluation: Committee Member 4/Membre de comité 4
Name of Applicant/Nom du chercheur: Archie, Suzanne
Application No./Numéro de demande: 454732
Agency/Agence: CIHR/IRSC
Competition/Concours: 2020-11-19 Catalyst Grant: Cannabis and Mental Health/Subvention catalyseur : Cannabis et santé mentale
Committee/Comité: Catalyst Grant: Cannabis and Mental Health/Subvention de catalyseur: Cannabis et santé mentale
Title/Titre: Insights about Cannabis and Psychosis: How do young people with early psychosis conceptualize the link between cannabis and psychosis, particularly those from Black racialized backgrounds?

Assessment/Évaluation:

1. Research Approach

a) Clarity, quality and appropriateness of the research design (i.e., rationale, questions, approach and methodology).

The current project aims to explore the understanding of young people with early psychosis and cannabis use disorder on the impact of cannabis on psychosis experience. This will be carried through out through the use of the Back to Reality Video Game SERIES. The SERIES could have an impact on the delivery of EPI care to patients with FEP and CUD by increasing insight about psychosis and cannabis use.

Overall, this is a well-thought-out proposal with clearly stated rationale and objectives. Specifically, it aims to establish the following:

1: Examine perceptions of the mental health effects of cannabis on psychosis
2: Establish the feasibility of knowledge acquisition [about mental health effects of cannabis on psychosis] comparing the SERIES versus a control game.
3: Develop a strong participatory and community engagement component

A mixed method approach will be used to reach objectives utilizing SERIES, which has received previous funding for its development, and the current project represents a logical continuation.

The proposed method seems highly appropriate for the research to be undertaken. Quantitative analyses are based on previous findings with well-argued required sample sizes for the present study.

b) Completeness of the literature review and relevance to study design/research plan.
Novel strategies are needed, and this is well-argued. Previous experience with SERIES showed improvements in knowledge about cannabis and psychosis and was experienced an enjoyable educational tool by participants. There is a strong rationale for the benefits of such a more culturally grounded approach, especially among racialized groups experiencing more adverse routes to EPI care.
c) Feasibility of the research approach (including recruitment of subjects, project timeline, preliminary data where appropriate, etc.) to address the research questions.
The project has qualified leads from five different sites, with a recruitment approach that has previously been implemented successfully.

d) Extent to which the perspectives of lived experience is meaningfully included in all stages of research, including research design, execution, knowledge translation and dissemination, and evaluation.

This is a strong aspect of the current proposal with an advisory committee that consists those with lived experiences, including young people and family members. Many already have contributed to design and in

**Review Type/Type d'évaluation:**	Committee Member 4/Membre de comité 4
**Name of Applicant/Nom du chercheur:**	Archie, Suzanne
**Application No./Numéro de demande:**	454732
**Agency/Agence:**	CIHR/IRSC
**Competition/Concours:**	2020-11-19 Catalyst Grant: Cannabis and Mental Health/Subvention catalyseur : Cannabis et santé mentale
**Committee/Comité:**	Catalyst Grant: Cannabis and Mental Health/Subvention de catalyseur: Cannabis et santé mentale
**Title/Titre:**	Insights about Cannabis and Psychosis: How do young people with early psychosis conceptualize the link between cannabis and psychosis, particularly those from Black racialized backgrounds?

Assessment/Évaluation:

round table discussions which instills confidence that this aspect will be very well addressed.

e) Viability of proposed approach to mitigate potential conflict of interests and/or impact of industry relationships (if applicable) on research project.
No concerns
f) Quality and appropriateness of Sex- and Gender-Based analysis Plus (SGBA+) is appropriately integrated throughout the proposal, including study rationale, experimental design, methods, analysis, as well as data interpretation and reporting.
SGBA+ is of high quality and well-addressed throughout the proposal.
g) Quality of the proposed plan to include consideration of specific populations (e.g., ethnicity, socioeconomic, sex, gender, minorities such as official language minority populations) and health equity.
Same, and fully addressed as an inherent part of the proposal.
h) Anticipation of difficulties that may be encountered in the research and plans for management.
Recruitment is raised a potential issue, but community outreach activities of the researchers, as well as the involvement of those with lived experience, alleviate these concerns to a significant extent.

2) Applicant(s)
a) Qualifications of the applicant(s), including training, experience and independence (relative to career stage)/ Experience of the applicant(s) in the proposed area of research and with the proposed methodology.
The NPI is Co-Chair Anti-Black Racism Task Force at her Department and past Clinical Director of the Cleghorn Early Psychosis Intervention Clinic. NPI has previously been involved with studies utilizing mixed methods, although perhaps not as the lead investigator with only one first-authored publications the past five years. Overall, however, this appears to be a good team with the background and expertise to carry out the research.
b) Ability to successfully and appropriately disseminate research findings, as demonstrated by knowledge translation activities (publications, conference presentations, briefings, media engagements, etc.).
NPI and team seem in a strong position to successfully disseminate research findings with already existing partnerships and dissemination activities.
3) Impact of the Research
a) Originality of the proposed research, in terms of the hypotheses/research questions addressed, novel technology/methodology, and/or novel applications of current technology/ methodology.
High.
b) Research proposal addresses a significant need or gap in mental health research and/or the mental health system.
Outcomes of results are stated realistically and address a significant and gap in mental health research.
d) Appropriateness and adequacy of the proposed plan for knowledge dissemination and exchange.
Knowledge translation efforts to be undertaken are promising. The investigators have a wide network that will contribute to the distribution of the SERIES across the country.

**Review Type/Type d'évaluation:**	Committee Member 4/Membre de comité 4
**Name of Applicant/Nom du chercheur:**	Archie, Suzanne
**Application No./Numéro de demande:**	454732
**Agency/Agence:**	CIHR/IRSC
**Competition/Concours:**	2020-11-19 Catalyst Grant: Cannabis and Mental Health/Subvention catalyseur : Cannabis et santé mentale
**Committee/Comité:**	Catalyst Grant: Cannabis and Mental Health/Subvention de catalyseur: Cannabis et santé mentale
**Title/Titre:**	Insights about Cannabis and Psychosis: How do young people with early psychosis conceptualize the link between cannabis and psychosis, particularly those from Black racialized backgrounds?

Assessment/Évaluation:

Open Science and Knowledge Translation Plan

a) Appropriateness and adequacy of the proposed plans for incorporating open science principles, practices and/or tools into their work.

No concerns.

b) Evidence in the Knowledge Translation Plan that applicants will actively engage in these activities for the duration of the grant, including their ability to successfully and appropriately disseminate research findings and related information such as protocols and data sets with the broader research community besides published manuscripts and abstracts

No concerns.

Review Type/Type d'évaluation: SO Notes /Notes de l'agent scientifique
Name of Applicant/Nom du chercheur: Archie, Suzanne
Application No./Numéro de demande: 454732
Agency/Agence: CIHR/IRSC
Competition/Concours: 2020-11-19 Catalyst Grant: Cannabis and Mental Health/Subvention catalyseur : Cannabis et santé mentale
Committee/Comité: Catalyst Grant: Cannabis and Mental Health/Subvention de catalyseur: Cannabis et santé mentale
Title/Titre: Insights about Cannabis and Psychosis: How do young people with early psychosis conceptualize the link between cannabis and psychosis, particularly those from Black racialized backgrounds?

Assessment/Évaluation:

Strengths:

The study aims to evaluate the use of a video game (SERIES) focusing on a black youth hero with psychosis and a cannabis use disorder to examine perceptions of effects of cannabis on mental health in black immigrant youth who have a new diagnosis of psychosis. The study aims to use the video game to communicate how cannabis use might be impacting on psychosis and evaluate the impact of the intervention in enhancing youth understanding of the link between cannabis and psychosis. Do they come to a better understanding of how cannabis could be impacting their psychosis through the intervention.
The study includes an elegant control condition, which was considered a strength.
Participatory action nature of the research to assure engagement and solid knowledge translation of this research with the target community was also considered a strength.
Perceived effects of cannabis on mental health is not well studied in black youth with immigrant backgrounds therefore considered important research. The game has been used with a number of high risk populations already as a way to communicate balanced information on cannabis and psychosis risk, which speaks to feasibility.
The proposal is well written and the protocol well-presented. The role and level of engagement of advisory council was well described. Feasibility was evaluated as being very high.

Team membership seemed appropriate for the proposed research and covered all three components of this research (psychosis, cannabis and black community engagement). Publication record of the PI was modest. But all team members appeared to fit well in the application.

The team seemed well positioned to conduct the study, and community-based letters of support were strong.

Impact was considered to be potentially high and knowledge translation plan was strong.

Weaknesses:

It was not clear why the researchers opted for qualitative pre-post game evaluation but did not incorporate a quantitative pre-post assessment.

[redacted]

**Review Type/Type d'évaluation:**	SO Notes /Notes de l'agent scientifique
**Name of Applicant/Nom du chercheur:**	Archie, Suzanne
**Application No./Numéro de demande:**	454732
**Agency/Agence:**	CIHR/IRSC
**Competition/Concours:**	2020-11-19 Catalyst Grant: Cannabis and Mental Health/Subvention catalyseur : Cannabis et santé mentale
**Committee/Comité:**	Catalyst Grant: Cannabis and Mental Health/Subvention de catalyseur: Cannabis et santé mentale
**Title/Titre:**	Insights about Cannabis and Psychosis: How do young people with early psychosis conceptualize the link between cannabis and psychosis, particularly those from Black racialized backgrounds?

Assessment/Évaluation:

There were concerns that some participants might have difficulty accessing the virtual platform given the diverse social backgrounds of the target population (e.g., sufficient broadband, access to devices needed to play the game).

Budget:

No concerns with the budget were raised

****

Note: The final rating of the application, provided in the Notice of Decision (NOD), is the averaged rating of the peer review committee members following the discussion of the application during the committee meeting, and therefore may differ from the ratings provided by the assigned reviewers in their respective reviews.

Remarque : La cote définitive de la demande, qui apparaît dans l'avis de décision, représente la moyenne des cotes accordées par les membres du comité d' évaluation par les pairs après avoir débattu de la demande à la réunion du comité. Elle peut donc différer de celle donnée par les évaluateurs dans leur évaluation respective.

````````````````````````````````````````````````````````````````````````````````````````````````````````````````````````
````````````````````````````````````````````````````````````````````````````````````````````````````````````````````````
